# Magnesium Sulfate as a Rescue Therapy for Refractory Unstable Supraventricular Tachycardia in the Emergency Department: A Case Report

**DOI:** 10.7759/cureus.106651

**Published:** 2026-04-08

**Authors:** Nordia Thompson-Newell, Michelle Gordon Taylor, Shuvra Dasgupta

**Affiliations:** 1 Emergency Medicine Division, University Hospital of the West Indies/University of the West Indies, Kingston, JAM

**Keywords:** anti-arrhythmic drugs, emergency cardiology, emergency medicine, magnesium sulphate, refractory arrhythmia, refractory svt, supraventricular tachycardia, unstable supraventricular tachycardia

## Abstract

Refractory unstable supraventricular tachycardia (SVT) that is unresponsive to both chemical cardioversion and electrical cardioversion is a rare phenomenon that may lead to significant morbidity and mortality. For this subcategory of patients, there are no clear recommendations in the current American Heart Association (AHA)/American College of Cardiology (ACC)/European Society of Cardiology (ESC) guidelines for their management. A 59-year-old male sought care in the emergency department due to chest pain, palpitation, shortness of breath, and pre-syncope. On evaluation, the patient had an unstable SVT as evidenced by acute heart failure on examination and an electrocardiogram demonstrating SVT. The SVT was unresponsive to both chemical (adenosine and amiodarone) and synchronized electrical cardioversion. With no other chemical cardioversion options and no clear recommended management guidelines, the patient was successfully converted with the use of 2 g intravenous magnesium sulfate. This case highlights the importance of considering unconventional therapy, such as magnesium sulphate, as a rescue agent in cases where the standard recommendations are ineffective, unavailable, or contraindicated.

## Introduction

Supraventricular tachycardia (SVT) is a common cardiac arrhythmia that presents to the emergency department (ED) [1]. SVT has an estimated prevalence of 2.25 per 1,000 persons and a higher incidence in women [2]. While often manageable, sustained SVT can lead to significant morbidity, including chest pain, heart failure, and syncope, particularly in patients with pre-existing cardiac compromise [3]. Current international guidelines provide a clear framework for managing stable and unstable SVT. First-line therapy begins with vagal maneuvers and progresses to intravenous adenosine if vagal maneuvers are unsuccessful [4,5]. Synchronized electrical cardioversion remains the definitive intervention for hemodynamically unstable patients or those refractory to pharmacotherapy [4,5]. Unstable SVT is defined by the advanced cardiac life support (ACLS) guidelines as those presenting with hypotension, signs of shock, ischemic chest pain, acute heart failure, and altered mental status [4]. 

Though the majority of patients convert with standard therapy, clinicians occasionally face challenging clinical scenarios such as SVT that persists despite multiple rounds of chemical and electrical cardioversion. This refractory state presents a critical dilemma, as prolonged tachycardia risks precipitating cardiogenic shock that demands urgent, alternative treatment strategies [6]. Refractory SVT is defined as "SVT that doesn't convert to sinus rhythm despite the administration of two doses of adenosine at or above the American Heart Association (AHA) recommended doses; and often requires a second drug (amiodarone, esmolol or procainamide) for treatment" [4]. In some cases, despite using a recommended second drug and synchronized cardioversion, patients still remain in "refractory SVT." Current guidelines from the AHA, American College of Cardiology (ACC), and European Society of Cardiology (ESC) have no clear recommendations for this subgroup of refractory unstable SVT. In these dire circumstances, intravenous magnesium sulfate (MgSO₄) has emerged as a potential rescue option [7].

Published evidence, drawn largely from case reports and small series, indicates that intravenous MgSO₄ can effectively terminate refractory SVT. Additionally, MgSO₄ offers a favorable safety profile with a minimal risk of hypotension [3,7]. On this background, MgSO₄ may be considered as a last resort therapy in select, high-risk cases that are unresponsive to standard treatment. We report the case of a patient with hemodynamically unstable, refractory SVT that was successfully terminated with intravenous MgSO₄. This was after failed responses to synchronized electrical cardioversion and standard antiarrhythmic medications. This case aims to reinforce the evidence for MgSO₄ as a viable, “last resort” life-saving intervention in the emergency management of refractory unstable SVT.

## Case presentation

A 59-year-old male with no known medical conditions presented to the ED with a three-hour history of sudden onset chest pain, difficulty breathing, palpitations, and a near syncopal episode. He denied syncope, cough, lower limb swelling, orthopnea, paroxysmal nocturnal dyspnea, or vomiting. 

**Figure 1:**
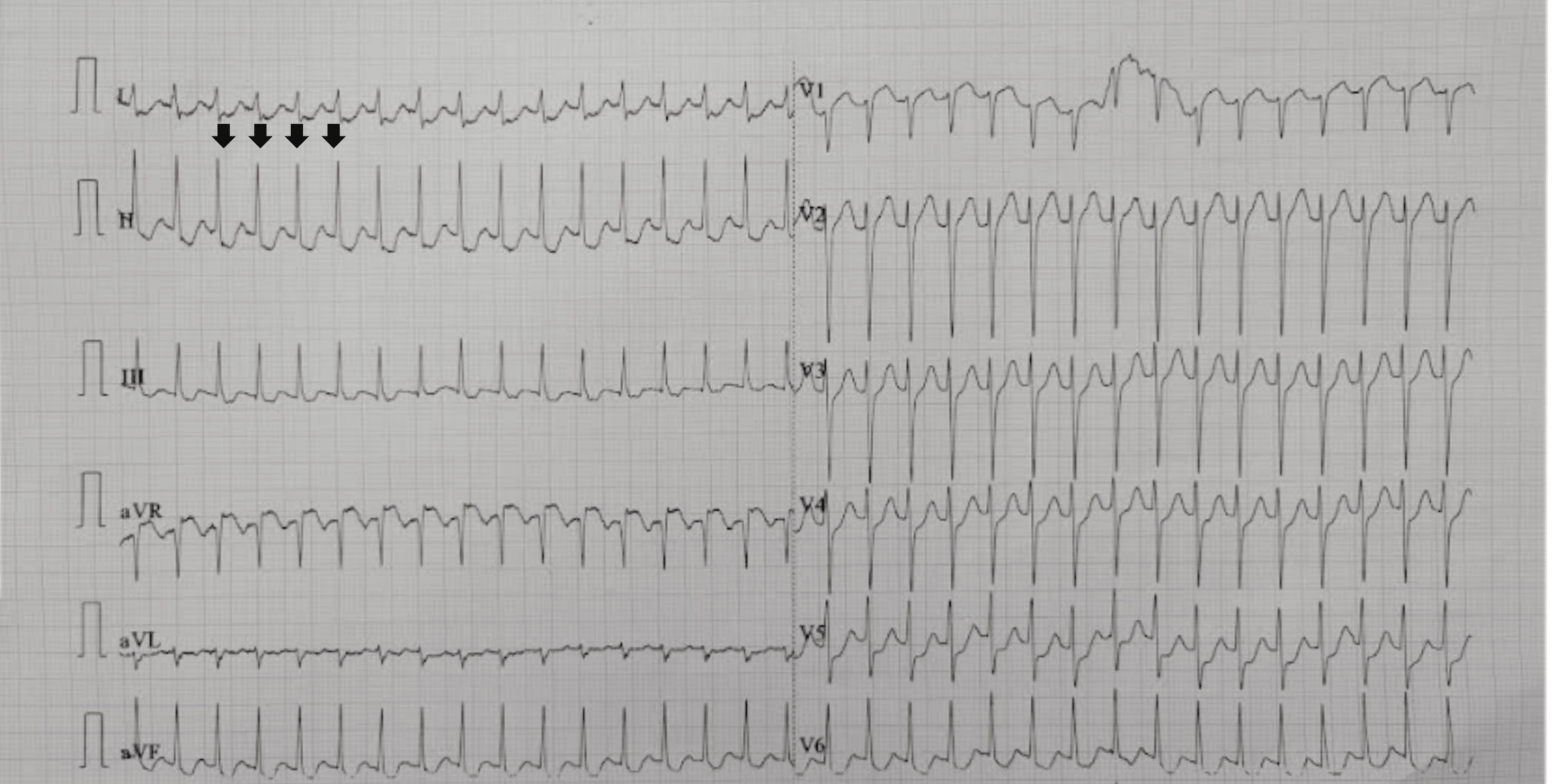
The initial ECG demonstrating supraventricular tachycardia at a rate of 198 beats per minute (arrow: narrow QRS complexes with no preceding p wave)

On arrival, he was alert but in moderate respiratory distress, diaphoretic, cool, and clammy. Vital signs included HR 199 beats per minute (bpm), RR 40, BP 123/79 mmHg, and SpO₂ 90% on room air. He received oxygen via a face mask. Cardiovascular exam revealed jugular venous distension (JVD) and an apical rate of 200 bpm. Respiratory exam showed bilateral basal crepitations and expiratory wheeze. The patient had no history of pulmonary disease (including asthma or chronic obstructive pulmonary disease). His ECG demonstrated SVT at 198 bpm (Figure 1). This finding, along with the patient's clinical signs of acute heart failure, confirmed the patient's hemodynamic instability. An additional differential considered was acute coronary syndrome. Of note, an SVT subtype could not be determined on the patient's emergency presentation. The patient received two large-bore intravenous accesses, and laboratory investigations were done, including complete blood count, urea and electrolytes, prothrombin time, partial thromboplastin time, international normalized ratio, and troponin. The blood investigations revealed an elevated creatinine 129 µmol/L (reference range: 53-115 µmol/L) and a low-normal magnesium 0.96 mmol/L (reference range 0.64-1.06 mmol/L). The troponin was negative. All other blood investigations were unremarkable.

**Figure 2:**
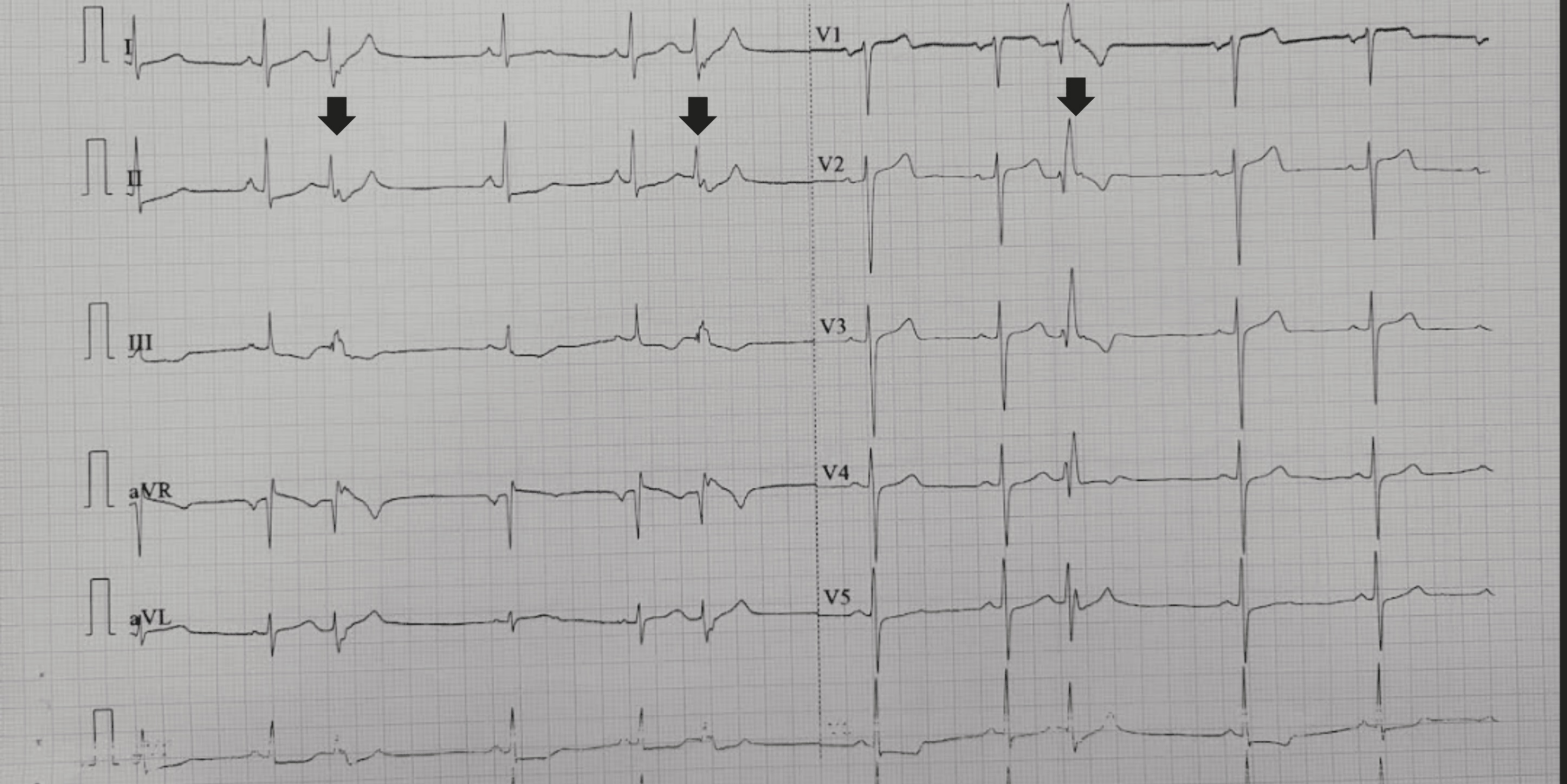
ECG during magnesium sulphate infusion demonstrating sinus rhythm with ventricular trigeminy at a rate of 66 beats per minute (arrow: ventricular ectopics)

**Figure 3:**
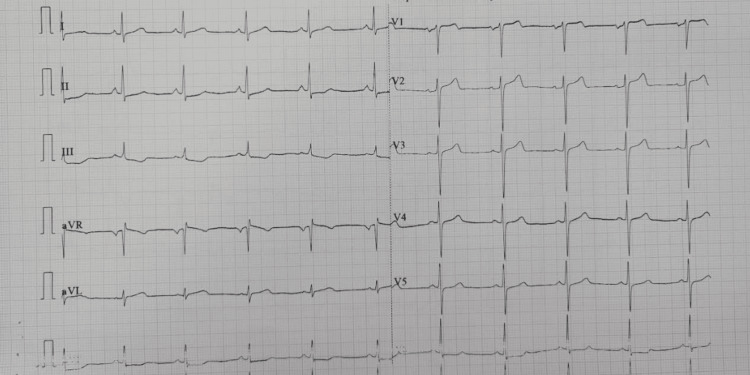
ECG post magnesium sulfate infusion showing sinus rhythm at a rate of 66 beats per minute

The patient gave informed consent for his ED management. Adenosine (6 mg, then 12 mg) failed. Synchronized electrical cardioversion was attempted at 100 J, 150 J, and 200 J after sedation (morphine 5 mg intravenously (IV) and midazolam 5 mg IV), with only brief sinus rhythm conversion. A subsequent IV amiodarone load (150 mg) and infusion (1 mg/minute) were initiated, but SVT persisted at 180 bpm after one hour of the infusion. Other recommended agents were considered: procainamide was unavailable, and beta-blockers and calcium channel blockers were contraindicated in the setting of acute heart failure [4]. IV MgSO₄ (2 g over 20 minutes) was administered and the rhythm converted to ventricular trigeminy (Figure 2), then bigeminy, and finally to sinus rhythm (70 bpm) within 15 minutes (Figure 3), with symptomatic improvement. The patient had no significant adverse effects during and after the administration of MgSO₄.

Based on the nature of the patient's chest pain and the negative troponin levels, the patient was treated for unstable angina and received aspirin 325 mg chewed, clopidogrel 300 mg orally, and IV unfractionated heparin 4000 units stat then 1000 units per hour (standard therapy). He also received IV furosemide 40 mg to treat the acute heart failure, as part of his total management. He was then admitted to Cardiology. During his admission, he had a transthoracic echocardiogram (TTE), which showed moderate to severe left ventricular hypertrophy (LVH) with normal left ventricular systolic function and no wall motion or valvular abnormalities. Throughout his admission, he maintained sinus rhythm and was discharged after four days for outpatient coronary angiography and planned catheter ablation to prevent recurrent SVT episodes.

## Discussion

This case report describes a rare case of unstable SVT that was resistant to synchronized electrical cardioversion and standard drug therapy. SVT is a common arrhythmia seen in the ED, with a conversion success rate of 98% with vagal maneuver, pharmacological management, or both [4]. Stable SVTs that are refractory to adenosine or unstable SVTs are treated with synchronized cardioversion [3,5], with a success rate of over 80% [8]. The 2025 ACLS guidelines suggest that if the patient is unresponsive to synchronized cardioversion, after three shocks, other antiarrhythmics should be considered, such as amiodarone [4]. There is sparse data to support the use of additional therapy in cases where the SVT is refractory to adenosine, escalated doses of biphasic synchronized cardioversion, and amiodarone or procainamide infusion. Of note, the AHA/ACC has no clear recommendation for this subset of refractory SVTs [3]. The index case was administered sequential doses of adenosine (6 mg followed by 12 mg), escalated to synchronized cardioversion, and an amiodarone infusion, all of which failed to achieve conversion. Subsequent discussion of pharmacologic alternatives ensued. Procainamide was unavailable institutionally. Beta-blockers and calcium channel blockers were accessible but were contraindicated due to the presence of heart failure signs and an undetermined ejection fraction. MgSO₄ infusion, though unconventional, converted the patient’s unstable, refractory SVT.

Magnesium serves as a predominantly intracellular cation in humans, with a mere 0.3-2% of the body's total magnesium found within the blood serum [9]. Homeostatic mechanisms work to preserve normal serum levels, even at the cost of depleting intracellular magnesium stores [9]. Consequently, serum magnesium is often a poor surrogate for total body magnesium status [10]. Past research indicates that intracellular magnesium deficiency can coexist with normal serum levels [10]. Importantly, correcting this deficiency through magnesium administration has been shown to reduce the frequency of cardiac arrhythmias in affected patients [10]. This concept is further supported by a randomized double-blinded study involving patients undergoing catheter ablation, which found that 89% of patients with normal serum magnesium had low intracellular levels [11]. The same study demonstrated that an intravenous magnesium infusion promptly corrected this intracellular deficit [11]. Notably, an independent body of evidence links low intracellular magnesium to an increased risk of various cardiac arrhythmias, including SVT [11,12]. This relationship is clinically nuanced, as a systemic magnesium deficiency can indeed be present despite unremarkable serum levels. MgSO₄’s antiarrhythmic properties are thought to derive from its function as a natural calcium channel blocker, which modulates atrioventricular (AV) nodal conduction, and its role as an essential cofactor for the myocardial sodium-potassium adenosine triphosphatase (ATPase) pump [13]. In the index case, this pathogenesis warrants strong consideration as a contributing factor for the refractory SVT, particularly given the excellent and definitive response to intravenous MgSO₄.

The electrophysiological effects of magnesium on the supraventricular conduction pathway have been well described. It includes the following: decreased automaticity, increased sinus atrial node recovery time, increased intra-atrial and AV conduction and refractory period, and blocks antegrade and retrograde conduction within accessory pathways [14]. This effectively restores the sinus rhythm in these patients [12]. Importantly, research has shown that the effectiveness of magnesium in patients with SVT depends on the subtype of tachycardia [12,15]. Specifically, MgSO₄ showed a greater conversion rate in patients with a dual AV conduction [12,15]. Hence, this may be a factor contributing to its inconsistent impact and the superiority of other drugs, such as adenosine, for the treatment of most SVTs [12]. Notably, the detection of the specific subtype of SVT is difficult with a 12-lead ECG only, and the subtypes are best diagnosed with electrophysiological studies [16]. Electrophysiological studies were not readily available at the facility where the patient was seen; hence, the specific subtype was not determined. With MgSO₄’s good safety profile and cost effectiveness [17] and evidence supporting its use for this rare presentation of SVT [12,15], it is prudent that magnesium is considered in patients with refractory unstable SVT. Importantly, though, it may only be considered as a “last resort” in the few high-risk cases and not as an alternative to standard therapy.

## Conclusions

This case highlights a rare presentation of chemical and electrical cardioversion refractory unstable SVT, and demonstrates the complexity of managing these cases. The patient had prompt recognition of SVT and initiation of management as per the ACLS guidelines. However, despite utilizing recommendations including adenosine, synchronized electrical cardioversion, and intravenous amiodarone, the patient had persistent refractory SVT complicated by acute heart failure. The patient successfully converted to sinus rhythm with the administration of intravenous MgSO₄ and had no adverse events. While MgSO₄ is not included in the standard treatment for SVTs, there is evidence to support its use in a select high-risk group of refractory unstable SVT cases. The rapid efficacy and safety in this case further emphasize MgSO₄’s potential role in this subgroup of patients. This case underscores the importance of considering MgSO₄ as a salvage pharmacological therapy in cases where SVT is refractory to standard treatment. Also, the case highlights consideration in patients who may have contraindications to drugs, such as beta-blockers and calcium channel blockers, and in settings where agents, such as procainamide, are unavailable. Further research and case reports would be beneficial to guide the management of this rare clinical subset of patients.
